# Commentary: Zika Virus: the Latest Newcomer

**DOI:** 10.3389/fmicb.2016.01028

**Published:** 2016-06-28

**Authors:** Adam T. Craig, Beverley J. Paterson, David N. Durrheim

**Affiliations:** School of Medicine and Public Health, University of NewcastleCallaghan, NSW, Australia

**Keywords:** epidemiology, surveillance, disease surveillance, Zika virus (ZIKV), outbreaks, communicable diseases, emerging, communicable disease epidemiology

We commend Saiz et al. (April 19) (Saiz et al., 2016) for their contribution to the growing body of evidence linking Zika virus infection and Guillain-Barré syndrome (GBS). With 13 countries or territories now reporting increased incidence of GBS and/or laboratory confirmation of Zika virus infection in GBS cases (World Health Organization, 2016b) the authors' contribution is timely and builds the case for heightened vigilance for the detection and response to Zika virus outbreaks using syndromes associated with infection or its complications.

Saiz et al. note the surge in recently reported GBS cases, temporally and geographically linked with Zika virus infection, highlighting a near 20-fold increase in case numbers following the French Polynesia outbreak (Oehler et al., 2014). In their paper the authors neglect to discuss the potential effect enhanced surveillance, currently being implemented by many Zika-affected and at-risk countries, may have on data collected, data comparability and the situational “picture” presented.

An example of the effect of enhanced surveillance following a high profile outbreak comes directly from the Global Polio Eradication Initiative (Global Polio Eradication Initiative, 2016). In 2015 countries that were either wild polio virus endemic (Afghanistan and Pakistan) or most recently endemic (Nigeria and India), where there is clearly intensive surveillance, rates of Acute Flaccid Paralysis (AFP)—the surveillance marker indicator for polio surveillance and the most common caused by GBS (Olivé et al., 1997; World Health Organization, 2014)—detection was up to 18 times higher than expected when compared with the average performance of countries in the same World Health Organization (WHO) Regions. (Craig et al., 2016; World Health Organization, 2016a).

While it is difficult to quantify what proportions of the noted increases in GBS/AFP cases are (i) due to actual excess case occurrence associated with recognized Zika virus outbreaks or (ii) the result of frenzied case finding where previously surveillance slumbered, caution should be exercised in assuming that these increases mirror the scale of Zika virus epidemics. The greater utility may be in considering trends before surveillance is enhanced once local occurrence of illness is confirmed. Further research to quantify the effect of enhanced surveillance in relation to the ongoing Zika situation, and during other public health emergencies, is necessary.

## Author contributions

AC, BP, and DD conceived the ideas presented in the article. AC led the drafting of the article with significant input from BP and DD. AC led the submission process.

### Conflict of interest statement

The authors declare that the research was conducted in the absence of any commercial or financial relationships that could be construed as a potential conflict of interest.
